# GPX4 Inhibition Enhances the Antitumor Effect of PARP Inhibitor on Homologous Recombination Proficient Ovarian Cancer Cells

**DOI:** 10.2174/0115680096334278240821100404

**Published:** 2024-08-26

**Authors:** Jiaxin Gu, Senmi Qian, Fangfang Qian, Xiaodong Wu, Lifeng Chen, Xiaojing Chen, Zhuoye Chen, Feifei Song, Mengxia Zheng, Lingfang Wang, Xiaodong Cheng

**Affiliations:** 1 Department of Gynecologic Oncology, Women's Hospital, School of Medicine, Zhejiang University, Hangzhou, Zhejiang, China;; 2 Zhejiang Key Laboratory of Precision Diagnosis and Therapy for Major Gynecological Diseases, Hangzhou, Zhejiang, China;; 3 Department of Gynecology, Hangzhou Third People's Hospital, Hangzhou, Zhejiang, China;; 4 Department of Gynecology, the First Affiliated Hospital, School of Medicine, Zhejiang University, Hangzhou, Zhejiang, China

**Keywords:** Ovarian cancer, GPX4, PARP inhibitors, homologous recombination deficiency (HRD) combination therapy, ROS

## Abstract

**Background:**

Poly (ADP-ribose) polymerase inhibitors (PARPi) are now widely used in BRCA1/2 mutation or homologous recombination (HR) deficiency ovarian cancer but have limited efficacy in HR-proficient patients. GPX4 is a key regulator of ferroptosis and has been proven to be associated with multiple drug sensitivities. As a molecule that regulates the sensitivity of multiple drugs, the relationship between GPX4 and the efficacy of PARPi in HR-proficient ovarian cancer has not been elucidated.

**Methods:**

In this study, siRNA transfection was used to regulate the expression of GPX4. The effect of GPX4 inhibition on HR-proficient ovarian cancer was determined by CCK-8 assay and flow cytometry. Immunofluorescence and comet assay were used to reflect DNA damage. ROS production was measured using DCFH-DA and flow cytometry. The combination index of PARP inhibitors and RSL3 was calculated using CompuSyn software based on Chou-Talalay methodology.

**Results:**

GPX4 inhibition confers HR-proficient ovarian cancer cells sensitive to PARPi due to ROS generation and oxidative stress caused DNA double-strand breakage. The combination of olaparib and niraparib with GPX4 inhibitor RSL3 also showed a synergistic effect.

**Conclusion:**

Combining GPX4 inhibition with PARP inhibitors resulted in a notable increase in DNA damage, ultimately causing the death of cancer cells with proficient HR pathways. Our findings may provide new therapeutic options for HR-proficient patients to benefit from PARP inhibitors and improve outcomes.

## 1 INTRODUCTION

In recent years, the use of PARP inhibitors has brought significant changes in the treatment of ovarian cancer. After ovarian cancer patients have achieved complete and partial remission through initial treatment or platinum-sensitive relapse treatment, the use of PARP inhibitors can significantly prolong Progression-Free Survival (PFS). However, for ovarian cancer with normal homologous recombination repair function, the efficacy of PARP inhibitors is still very limited [1]. Therefore, finding methods to improve the efficacy of PARP inhibitors in HRD-negative patients is of great significance for treatment.

Combination therapy is considered an effective way to sensitize PARP inhibitors and overcome drug resistance. There are many ongoing clinical trials aimed at exploring the role of PARP inhibitor combination therapy in solid tumors. The combination strategies of PARP inhibitors mainly include traditional chemotherapy drugs, radiation, targeted agents, and immunotherapeutics [2]. For example, maintenance therapy with olaparib plus bevacizumab improves progression-free survival in advanced ovarian cancer patients [3]. In pancreatic cancer, the synergic effects of immunotherapy and tyrosine kinase inhibitors with PARPi, such as cediranib and pembrolizumab [4], have been investigated. In gastric cancer, ongoing clinical trials are testing the combination of PARPi with chemotherapy, immune checkpoint inhibitors, and anti-VEGF monoclonal antibodies [5]. However, there are still many problems that need to be solved, such as excessive drug toxicity caused by combined drugs. Clinical trials evaluating the combination of olaparib and chemotherapy terminated due to drug toxicity [6]. Many combined strategies lack accurate molecular markers that can be used to classify patients and select the most appropriate treatment.

As a key molecule of ferroptosis, GPX4 is a phospholipid hydrogen peroxide glutathione peroxidase (PHGPx). Multiple studies have reported that GPX4 can mediate the sensitivity of chemotherapeutic agents. Inhibiting GPX4 can enhance the efficacy of cisplatin in tumors, such as prostate cancer [7] and brain metastatic lung cancer [8]. Drug-resistant cells with high expression of Wnt receptor Frizzled7 (FZD7) are highly correlated with the upregulation of GSH metabolic pathways, including GPX4 [9]. GPX4 may also be used as a new biomarker for predicting efficacy and prognosis in patients, who received neoadjuvant chemotherapy [10]. At present, there are also studies indicating that PARP inhibitors can promote ferroptosis by inhibiting SLC7A11, and the combination of ferroptosis inducers and olaparib can produce a synergistic effect [11]. Arsenic trioxide synergizes with olaparib by promoting ferroptosis [12]. Bone metastatic breast cancer has developed olaparib resistance through increased glutamine uptake and GPX4 upregulation [13].

As a molecule that regulates the sensitivity of multiple drugs, the relationship between GPX4 and the efficacy of PARP inhibitors in HR-proficient ovarian cancer has not been elucidated. It was found that downregulating GPX4 in HR-proficient ovarian cancer cells can enhance the sensitivity of PARP inhibitors. The result suggests a possible synergistic combination therapy involving both PARP inhibitors and GPX4 inhibitors for HR-proficient patients.

## 2 MATERIALS AND METHODS

### 2.1 Cell Culture

A2780 and OVCAR8 were purchased from the American Type Culture Collection (ATCC, Manassas, VA, USA). Cells were cultured in RPMI 1640 (Gibco, USA) containing 10% fetal bovine serum (Gibco, USA). The incubator was set to 37°C with 5% CO2 and suitable humidity. Then, 1% penicillin/streptomycin (Gibco, USA) was added to the medium.

### 2.2 Reagent

Olaparib (HY-10162), niraparib (HY-10619), RSL3 (HY-100218A), and DCFH-DA (HY-D0940) were purchased from MedChemExpress (MCE). Olaparib and niraparib were prepared at 100 mmol/L, while RSL3 and DCFH-DA were prepared at 10 mmol/L in DMSO (Sigma, D2660) and stored at -80°C.

### 2.3 Cell Viability Assay and Drug Combination Analysis

Cells were seeded in 96-well plates and exposed to specified drug concentrations for 72 hours. Cell viability was then determined using the CCK-8 assay (Yeasen, 40203ES). The interaction between olaparib, niraparib, and RSL3 was evaluated using the CompuSyn software to determine if the combination had a synergistic effect, indicated by combination indexes (CI) <1.

### 2.4 siRNA Transfection

GPX4 and control siRNA were synthesized by GenePharma (Shanghai, China). Transient transfection was performed using DharmaFECT Transfection Reagents (Thermo), following the standard protocol. siRNAs used were:

Ctrl siRNA: 5’- UUCUCCGAACGUGUCACGU -3’

siGPX4 -1: 5’-GGAAGUGGAUGAAGAUCCA -3’

siGPX4 -2: 5’- GGAGUAACGAAGAGAUCAA-3’

### 2.5 Cell Apoptosis Measurement

Annexin V-FITC/PI apoptosis kit (Yeasen, 40302ES) was used to detect the percentage of cell apoptosis. Cells were seeded in six-well plates and subsequently treated with DMSO, olaparib (16μM), and niraparib (8μM) for 48 hours. Then, they were resuspended in a pre-chilled 1 x binding solution, followed by a staining process according to the kit instructions. All operations were performed in dark conditions, and the results were analyzed using a flow cytometer (CytoFLEX S, Beckman).

### 2.6 Immunofluorescence

Cells were seeded on sterilized coverslips, maintaining an optimal density, and were incubated for 24 hours. Then the cells underwent treatment with DMSO, olaparib (16μM), and niraparib (8μM) for another 24 hours. Following this, the cells were fixed using 4% paraformaldehyde, permeabilized with 0.1% TritonX-100, and then blocked using 5% bovine serum. Subsequently, the cells were incubated with γ-H2AX (Abcam, ab26350, 1:500) at 4 °C overnight. Afterward, they were stained with a secondary antibody (Invitrogen, A32723, 1:1000). DAPI (Abcam, ab104139) was used for counterstaining. Images were captured using a confocal microscope (STELLARIS5, Leica).

### 2.7 Quantitative Real-Time PCR

TRIzol reagent (Invitrogen, 15596018) was used to extract total RNA, and mRNA was converted to cDNA through the HiScript III RT SuperMix (Vazyme, R323-01). Quantitative real-time PCR was conducted using ChamQ Universal SYBR qPCR Master Mix (Vazyme, Q711-02) in tandem with the Fast Real-Time PCR System (LightCycler 480 II, Roche). The primers used are presented below: human GPX4: 5′- GCCTTCCCGTGTAACCAGT -3′(forward), 5′- GCGAACTCTTTGATCTCTTCGT -3′ (reverse), human PARP1: 5′-CGGAGTCTTCGGATAAGCTCT-3′ (forward), 5′- TTTCCATCAAACATGGGCGAC-3′ (reverse), human β-actin: 5′-TGGTATCGTGGAAGGACTC-3′ (forward), 5′-AGTAGAGGCAGGGATGATG-3′ (reverse).

### 2.8 Western Blot

Protein was extracted using RIPA buffer (Solarbio, R0010), and concentrations were standardized. Then, 20μg of each protein sample was separated on SDS-PAGE gels and then transferred onto NC membranes. These membranes were blocked using 5% milk at room temperature. They were incubated at 4°C with primary antibodies overnight, including γH2AX (Abcam, ab26350, 1:1000), PARP1 (Santa Cruz, sc8007, 1:1000), PARP (CST, 9542S, 1:2000), β-actin (Fude Bio, FD0060, 1:5000), GPX4 (Abcam, ab125066, 1:1000), and H3 (Proteintech, 17168-1-AP, 1:2000). Next day, membranes were incubated with HRP-linked secondary antibody, followed by visualization through imaging systems.

### 2.9 ROS Measurement

ROS production was measured using DCFH-DA, dissolved in DMSO, and diluted to working concentrations. Cells were seeded in 6-well plates and treated with DMSO, olaparib (16μM), and niraparib (8μM) for 48 h. Then, cells were incubated in 5μM DCFH-DA at 37 °C for 30 min. The fluorescence signal was measured using flow cytometry at 488 nm/525 nm. The images of cells were taken by laser scanning microscopy (DMi8L, Leica).

### 2.10 Comet Assay

Comet assay was performed according to the protocol (Abcam, ab238544). Cells were seeded in six-well plates and then treated with DMSO, olaparib (16μM), and niraparib (8μM) for another 24h. The comet agarose was pipetted onto the slide, and after the base layer had formed, the pipetted agarose/cell mixture was pipetted onto the top of the base layer. Then, cells were treated with a lysis buffer and an alkaline solution. The slides were placed in the electrophoresis chamber, which was run for 30 minutes at a voltage of 1V/cm. The slides were washed with water three times and then soaked in 70% alcohol for 5 minutes. Once the alcohol evaporated, they were stained with Vista green. Images were captured by fluorescence microscopy using a FITC filter (STELLARIS5, Leica). CometScore 2.1 Software (Tritek) was used to measure the average “DNA in tails (%)” of 40 cells to assess DNA damage.

### 2.11 Statistical Analysis

The experiments were carried out with three biological replicates. The data was presented as mean ± standard deviation (SD) and analyzed using GraphPad Prism 9.0. Statistics were considered significant at *p < 0.05, **p < 0.01, and ***p < 0.001.

## 3 RESULTS

### 3.1 GPX4 Inhibition Increases Sensitivity to PARP Inhibitor in HR-Proficient Ovarian Cancer

**Fig. (1):**
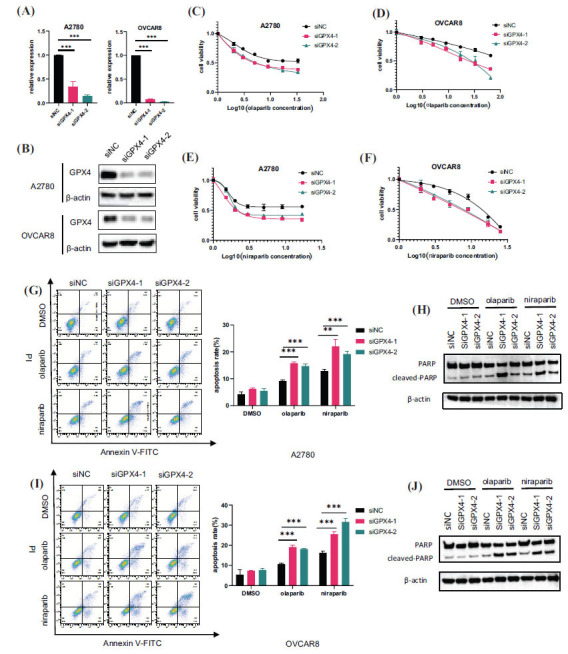
GPX4 inhibition increases sensitivity to PARP inhibitor in HR-proficient ovarian cancer. (**A**) GPX4 siRNAs were stably transfected into A2780 and OVCAR8 cells. qPCR was used to determine GPX4 RNA levels. (**B**) Western blot was used to determine GPX4 protein levels. **C**, **D**, **E**, **F**) The CCK8 assay was performed to detect cell viability in A2780 and OVCAR8 cells treated with olaparib and niraparib for 72 h. (**G, I**) Flow cytometry assay was performed to detect cell apoptosis in A2780 and OVCAR8 cells treated with olaparib (16μΜ) and niraparib(8μM) for 48h. (**H**, **J**) The expression of cleaved PARP and PARP was detected after treatments of olaparib (16μΜ) and niraparib (8μM) for 48h by western blot.

To investigate whether GPX4 mediates PARP inhibitor sensitivity in ovarian cancer cells, GPX4 was knocked down using two siRNAs transfected into A2780 [14] and OVCAR8 [15]; both are HR-proficient cell lines. qPCR and western blot showed a high knockdown efficiency (Fig. **1A** and **B**). After the knockdown of GPX4, olaparib and niraparib were added to cells at different concentrations for 72 h. CCK-8 indicated that GPX4 knockdown increased sensitivity to both olaparib and niraparib (Figs. **1C**-**F**). The flow cytometry assay verified that after PARP inhibitor treatment, GPX4 knockdown increased the proportion of apoptotic cells (Fig. **1G**, **I**). Western blot detected the levels of cleaved PARP and PARP, a marker of apoptosis. The finding indicated that GPX4 inhibition increased cleaved PARP (Fig. **1H**, **J**). In summary, inhibition of GPX4 promoted sensitivity to PARP inhibitors in HR-proficient ovarian cancer cells.

### 3.2 GPX4 Inhibition Enhances PARPi-Mediated DNA Damage in Ovarian Cancer Cells

**Fig. (2):**
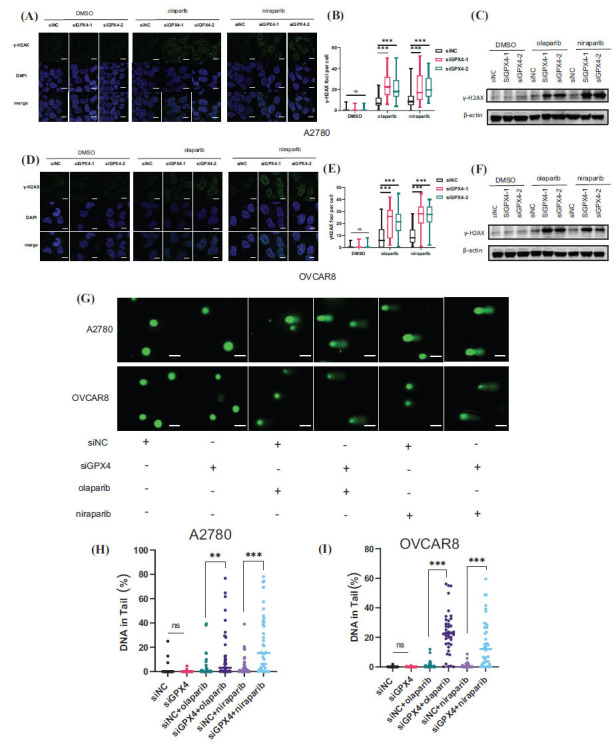
GPX4 inhibition enhances PARPi-mediated DNA damage in ovarian cancer cells. (**A**, **D**) Representative immunofluorescence images of γ-H2AX foci in cells after being treated with DMSO, olaparib (16μΜ), and niraparib (8μM) for 24h, scale bar = 10 μm. (**B**, **E**) Quantification of the numbers of γ-H2AX foci in each nucleus. Data was presented as mean ± SD from 50 cells. (**C**, **F**) Western blot showed the γ-H2AX protein levels after drug treatment. (**G**) Representative images of DNA damage in cells after treatment of DMSO, olaparib (16μΜ), and niraparib (8μM) for 24h, measured by comet assay, bar=50μm. (**H**, **I**) Quantified results by the tail moment in the comet assay data were presented from 40 cells.

Histone H2AX phosphorylation occurred on the four serine residues at the carboxyl terminus (producing γ H2AX). DNA double-strand breaks (DSBs) were sensitively detected by γH2AX. DNA damage levels were determined by the number of γH2AX foci within each cell. We detected significantly increased γH2AX foci per cell with GPX4 knockdown after olaparib or niraparib treatment compared to their control groups (Fig. **2A**, **D**). The numbers of γ-H2AX foci within each cell were counted (Fig. **2B**, **E**). Moreover, the results of the western blot suggested that GPX4 knockdown enhanced the protein expression levels of γ-H2AX (Fig. **2C**, **F**). According to the comet assay, GPX4 inhibition induced a higher proportion of DNA in the tail after PARP inhibitor treatment (Figs. **2G**, **H**, and **I**).

### 3.3 GPX4 Inhibition Enhances the DNA Damage of PARP Inhibitors by Inducing ROS Production

**Fig. (3):**
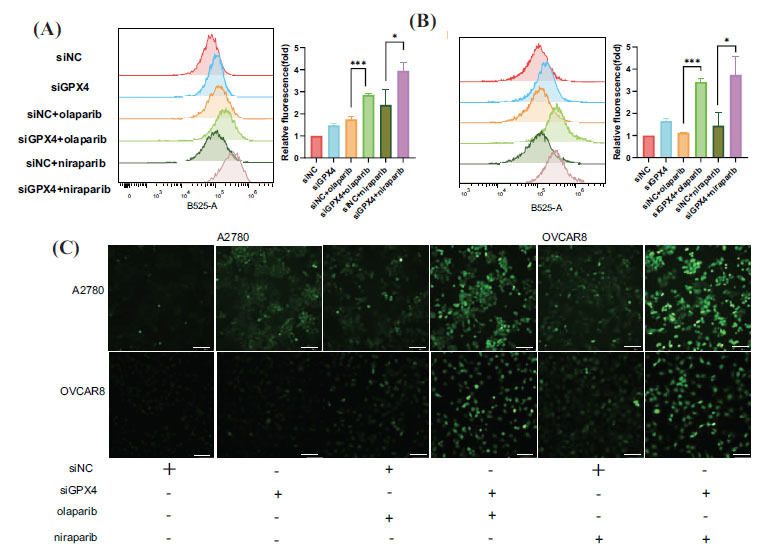
GPX4 inhibition enhances the DNA damage of PARP inhibitors by inducing ROS production. (**A**, **B**) Flow cytometry assay was performed to detect ROS levels in A2780 and OVCAR8 cells treated with olaparib (16μΜ) and niraparib (8μM) for 48h. (**C**) Representative images of ROS level in cells, bar = 100 μm.

Loss of GPX4 will subsequently lead to the accumulation of lipid-based reactive oxygen species (ROS) [16]. Vast ROS production can induce DNA damage and affect the DNA damage response (DDR) [17]. We detected whether GPX4 inhibition and PARP inhibitor treatment could increase the intracellular ROS levels in HR-proficient ovarian cancer cells. Cellular ROS were detected using a DCFH-DA probe. The flow cytometry results showed that GPX4 knockdown or PARP inhibitor treatment upregulated ROS levels individually. The GPX4 knockdown combined with the treatment of PARP inhibitors resulted in the highest ROS level (Fig. **3A**-**B**). ROS levels were also photographed using a fluorescence microscope, and the results were consistent with flow cytometry (Fig. **3C**).

### 3.4 PARP1 Trapping is not Involved in GPX4-Mediated Changes in PARP Inhibitors Efficacy

**Fig. (4):**
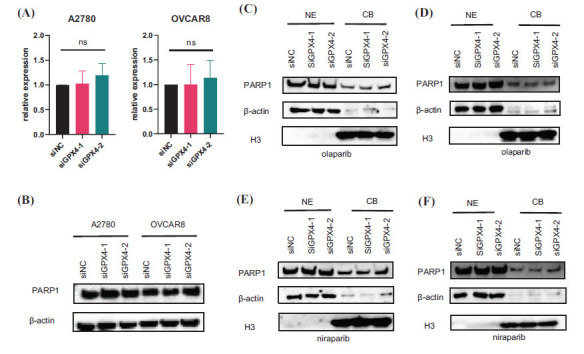
PARP1 trapping is not involved in GPX4-mediated changes in PARP inhibitor efficacy. (**A**) qPCR was used to determine PARP1 RNA levels. (**B**) Western blot was used to determine PARP1 protein levels. (**C-F**) The protein level of PARP1 in nuclear soluble and chromatin-bound fractions of GPX4 knockdown and control cells after olaparib and niraparib treatment was determined by Western blot. A2780 (**C**, **E**) OVCAR8 (**D**, **F**).

PARP inhibitors cause cell death by suppressing the activity of PARP [18]. Besides the inhibition of PARP catalytic activity, PARP inhibitors can trap PARP in DNA breaks and disrupt normal DNA damage repair. The cytotoxicity of trapped PARP-DNA complexes was greater than unrepaired SSBs caused by PARP inactivation [19]. So, we explored if GPX4 inhibition will influence the amount of PARP1 or PARP trapping ability. qPCR and Western blot demonstrated that GPX4 inhibition did not change PARP1 amount (Figure **4A**, **B**). We also observed no increased PARP1 protein trapped onto the chromatin in GPX4 inhibition cells compared to control cells (Figs. **4C**, **D**, **E**, **F**).

### 3.5 GPX4 Inhibitor RSL3 Synergistically Enhances the Antitumor Effect of PARP Inhibitors

**Fig. (5):**
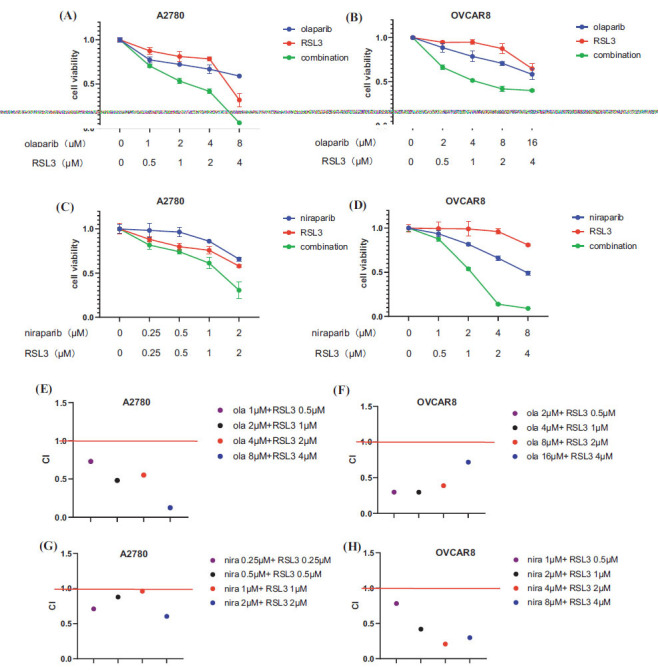
GPX4 inhibitor RSL3 synergistically enhances the antitumor effect of PARP inhibitor. (**A-D**) The CCK8 assay was performed to detect cell viability in A2780 and OVCAR8 cells treated with olaparib, niraparib, and RSL3 as indicated concentration for 72 h. (**E-H**) The Chou-Talalay plot showing the combination effect of indicated treatments.

In order to explore the synergistic impact of PARP inhibitors and GPX4 inhibitors, the viability of A2780 and OVCAR8 were examined (Figs. **5A**-**D**). These cells were treated with different concentrations of olaparib and niraparib in combination with RSL3, a widely used GPX4 inhibitor. To validate whether the synergy between PARP inhibitors and RSL3 was authentic and not merely additive or antagonistic, the combination index was determined using CompuSyn software, which utilizes the Chou-Talalay approach. The results revealed synergistic effects (CI < 1) in all groups (Figs. **5E**-**H**).

## 4 DISCUSSION

Ovarian cancer is the most lethally gynecological malignant tumor, has a hidden onset, and seriously endangers women's health. Among gynecological cancers, ovarian cancer is deadliest [20]. In newly diagnosed patients who received platinum-based chemotherapy and platinum-sensitive recurrences, PARP inhibitors are available as maintenance therapy. Inhibition of PARP activity leads to double-strand breaks from unrepaired single-strand breaks and DNA replication forks. About 50% of HGSOC tumors are homologous recombination defective, making it an important therapeutic target [1]. Among three available PARP inhibitors (olaparib, niraparib, and rucaparib) for maintenance therapy, olaparib is recommended in patients with BRCA mutations, while niraparib and rucaparib can be used irrespective of BRCA or HRD status. However, their benefit is lower compared with HRD patients [21]. Due to the unsatisfactory efficacy in HR-proficient ovarian cancer, many studies focus on expanding the application of PARP inhibitors. One of the important strategies to enhance PARP inhibitors’ efficacy is increasing DNA damage [22].

GPX4 is known as a key antioxidant enzyme, which plays a pivotal role in scavenging lipid peroxidation products and protecting cells from oxidative damage [23]. Loss of GPX4 activity leads to ROS accumulation [16]. In the present study, we found that olaparib and niraparib efficacy were significantly enhanced after GPX4 inhibition. Flow cytometry further confirmed that both GPX4 inhibition and olaparib, niraparib treatment were able to cause an increase of ROS in ovarian cancer cells. Such an effect could be synergistically enhanced by combination. By immunofluorescence staining and western blot analysis of γ-H2AX, we found that GPX4 inhibition increased PARPi-caused DSBs in HR-proficient cells. Therefore, we infer that oxidative DNA damage is one of the key mechanisms of GPX4 inhibition and PARPi synergy.

Mechanisms of PARPi include the inhibition of PARP enzyme activity and PARP trapping [19]. Here, we demonstrated that GPX4 inhibition did not change the amount of PARP1. Otherwise, in GPX4-inhibited cells, there was no increase in PARP1 protein trapped on chromatin after PARPi treatment.

A limitation of our study is that we lack *in vivo* experiments and clinical specimen validation. If our results include nude mouse subcutaneous tumors, organoids, or other models and verification of GPX4 level and PARP inhibitor sensitivity based on patient tissue, it will further support our conclusion.

In oncology, combination therapy has been tested to prolong PFS and overall survival (OS). Some research focused on combination therapy to complement the monotherapy of PARP inhibitors. For example, combining PARP inhibitors with MEK inhibitors [24] and ATR inhibitors [25] can exert synergistic effects and help to overcome PARPi resistance. In our study, it has been verified that the combination of PARP inhibitors and RSL3, a well-known GPX4 inhibitor, showed synergistic effects at different concentrations. However, most reported GPX4 inhibitors lack precise targets and will kill normal tissues and immune cells while killing tumor cells [26]. Therefore, our research is currently at the theoretical stage, and there is still a long way to go before entering clinical application. We hope that in the future, a highly selective GPX4 inhibitor with less toxic side effects will emerge for clinical application.

## CONCLUSION

Our study firstly found that GPX4 inhibition, combined with olaparib and niraparib, led to significant accumulation of DNA damage, promoting cancer cell death in HR-proficient ovarian cancer cells. Our findings may provide new therapeutic options for HR-proficient patients to benefit from PARP inhibitors and improve outcomes.

## Data Availability

All data generated or analysed during this study are included in this published article.

## LIST OF ABBREVIATIONS

ATCC: American Type Culture Collection
CCK-8: Cell Counting Kit-8
CI: Combination Index
DDR: DNA Damage Response
DMSO: Dimethyl Sulfoxide
DSBs: Double-Strand Breaks
GPX4: Glutathione Peroxidase 4
HGSOC: High-grade Serous Ovarian Carcinoma
HR: Homologous Recombination
HRD: Homologous Recombination Deficiency
OS: Overall Survival
PARPi: Poly (ADP-ribose) Polymerase Inhibitors
PFS: Progression Free Survival
ROS: Reactive Oxygen Species
siRNA: small interfering RNA

## References

[1] Konstantinopoulos P.A., Ceccaldi R., Shapiro G.I., D’Andrea A.D. (2015). Homologousrecombination deficiency: exploiting the fundamental vulnerability of ovariancancer.. Cancer Discov..

[2] Dréan A., Lord C.J., Ashworth A. (2016). PARP inhibitor combination therapy.. Crit. Rev. Oncol. Hematol..

[3] Ray-Coquard I., Pautier P., Pignata S., Pérol D., González-Martín A., Berger R., Fujiwara K., Vergote I., Colombo N., Mäenpää J., Selle F., Sehouli J., Lorusso D., Guerra Alía E.M., Reinthaller A., Nagao S., Lefeuvre-Plesse C., Canzler U., Scambia G., Lortholary A., Marmé F., Combe P., de Gregorio N., Rodrigues M., Buderath P., Dubot C., Burges A., You B., Pujade-Lauraine E., Harter P. (2019). Olaparib plus Bevacizumab as First-Line Maintenance in Ovarian Cancer.. N. Engl. J. Med..

[4] Di Federico A., Tateo V., Parisi C., Formica F., Carloni R., Frega G., Rizzo A., Ricci D., Di Marco M., Palloni A., Brandi G. (2021). Hacking Pancreatic Cancer: Present and Future of Personalized Medicine.. Pharmaceuticals (Basel).

[5] Ricci A.D., Rizzo A., Brandi G. (2021). DNA damage response alterations in gastric cancer: knocking down a new wall.. Future Oncol..

[6] Bendell J., O’Reilly E.M., Middleton M.R., Chau I., Hochster H., Fielding A., Burke W., Burris H. (2015). III Phase I study of olaparib plus gemcitabine in patients with advanced solid tumours and comparison with gemcitabine alone in patients with locally advanced/metastatic pancreatic cancer.. Ann. Oncol..

[7] Li M., Chen X., Wang X., Wei X., Wang D., Liu X., Xu L., Batu W., Li Y., Guo B., Zhang L. (2021). RSL3 enhances the antitumor effect of cisplatin on prostate cancer cells via causing glycolysis dysfunction.. Biochem. Pharmacol..

[8] Liu W., Zhou Y., Duan W., Song J., Wei S., Xia S., Wang Y., Du X., Li E., Ren C., Wang W., Zhan Q., Wang Q. (2021). Glutathione peroxidase 4‐dependent glutathione high‐consumption drives acquired platinum chemoresistance in lung cancer‐derived brain metastasis.. Clin. Transl. Med..

[9] Wang Y., Zhao G., Condello S., Huang H., Cardenas H., Tanner E.J., Wei J., Ji Y., Li J., Tan Y., Davuluri R.V., Peter M.E., Cheng J.X., Matei D. (2021). Frizzled-7 Identifies Platinum-Tolerant Ovarian Cancer Cells Susceptible to Ferroptosis.. Cancer Res..

[10] Sha R., Xu Y., Yuan C., Sheng X., Wu Z., Peng J., Wang Y., Lin Y., Zhou L., Xu S., Zhang J., Yin W., Lu J. (2021). Predictive and prognostic impact of ferroptosis-related genes ACSL4 and GPX4 on breast cancer treated with neoadjuvant chemotherapy.. EBioMedicine.

[11] Hong T., Lei G., Chen X., Li H., Zhang X., Wu N., Zhao Y., Zhang Y., Wang J. (2021). PARP inhibition promotes ferroptosis via repressing SLC7A11 and synergizes with ferroptosis inducers in BRCA-proficient ovarian cancer.. Redox Biol..

[12] Tang S., Shen Y., Wei X., Shen Z., Lu W., Xu J. (2022). Olaparib synergizes with arsenic trioxide by promoting apoptosis and ferroptosis in platinum-resistant ovarian cancer.. Cell Death Dis..

[13] Fan H., Xu Z., Yao K., Zheng B., Zhang Y., Wang X., Zhang T., Li X., Hu H., Yue B., Hu Z., Zheng H. (2024). Osteoclast Cancer Cell Metabolic Cross-talk Confers PARP Inhibitor Resistance in Bone Metastatic Breast Cancer.. Cancer Res..

[14] Xu J., Shen Y., Wang C., Tang S., Hong S., Lu W., Xie X., Cheng X. (2021). Arsenic compound sensitizes homologous recombination proficient ovarian cancer to PARP inhibitors.. Cell Death Discov..

[15] Kondrashova O., Topp M., Nesic K., Lieschke E., Ho GY., Harrell MI., Zapparoli GV., Hadley A., Holian R., Boehm E., Heong V., Sanij E., Pearson RB., Krais JJ., Johnson N., McNally O., Ananda S., Alsop K., Hutt KJ., Kaufmann SH., Lin KK., Harding TC., Traficante N. (2018). Australi an Ovarian Cancer Study (AOCS); deFazio A, McNeish IA, Bowtell DD, Swisher EM, Dobrovic A, Wakefield MJ, Scott CL. Methylat ion of all BRCA1 copies predicts response to the PARP inhibitor rucaparib in ovarian carcinoma. Nat Commun. 2018 Sep 28;9(1):3970.. Nat. Commun..

[16] Yang W.S., Stockwell B.R. (2016). Ferroptosis: Death by lipid peroxidation.. Trends Cell Biol..

[17] Srinivas U.S., Tan B.W.Q., Vellayappan B.A., Jeyasekharan A.D. (2019). ROS and the DNA damage response in cancer.. Redox Biol..

[18] Li H., Liu Z.Y., Wu N., Chen Y.C., Cheng Q., Wang J. (2020). PARP inhibitor resistance: the underlying mechanisms and clinical implications.. Mol. Cancer.

[19] Murai J., Huang S.N., Das B.B., Renaud A., Zhang Y., Doroshow J.H., Ji J., Takeda S., Pommier Y. (2012). Trapping of PARP1 and PARP2 by Clinical PARP Inhibitors.. Cancer Res..

[20] Siegel R.L., Miller K.D., Fuchs H.E., Jemal A. (2022). Cancer statistics, 2022.. CA Cancer J. Clin..

[21] Garg V., Oza A.M. (2023). Treatment of ovarian cancer beyond PARP Inhibition: Current and future options.. Drugs.

[22] Hockings H., Miller R.E. (2023). The role of PARP inhibitor combination therapy in ovarian cancer.. Ther. Adv. Med. Oncol..

[23] Xie Y., Kang R., Klionsky D.J., Tang D. (2023). GPX4 in cell death, autophagy, and disease.. Autophagy.

[24] Sun C., Fang Y., Yin J., Chen J., Ju Z., Zhang D., Chen X., Vellano C.P., Jeong K.J., Ng P.K.S., Eterovic A.K.B., Bhola N.H., Lu Y., Westin S.N., Grandis J.R., Lin S.Y., Scott K.L., Peng G., Brugge J., Mills G.B. (2017). Rational combination therapy with PARP and MEK inhibitors capitalizes on therapeutic liabilities in RAS mutant cancers.. Sci. Transl. Med..

[25] Kim H., Xu H., George E., Hallberg D., Kumar S., Jagannathan V., Medvedev S., Kinose Y., Devins K., Verma P., Ly K., Wang Y., Greenberg R.A., Schwartz L., Johnson N., Scharpf R.B., Mills G.B., Zhang R., Velculescu V.E., Brown E.J., Simpkins F. (2020). Combining PARP with ATR inhibition overcomes PARP inhibitor and platinum resistance in ovarian cancer models.. Nat. Commun..

[26] Li B., Cheng K., Wang T., Peng X., Xu P., Liu G., Xue D., Jiao N., Wang C. (2024). Research progress on GPX4 targeted compounds.. Eur. J. Med. Chem..

